# Study on the invitro synergistic susceptibility and biofilm inhibition mechanism of ceftazidime-avibactam combined with aztreonam against carbapenem-resistant *Klebsiella pneumoniae*

**DOI:** 10.3389/fmicb.2025.1542029

**Published:** 2025-03-20

**Authors:** Guangfen Wang, Hui Zhang, Qiaoping Wu, Jianqiang Xu, Xuedan Qiu, Jinyuan Chen, Fujie Cui, Jian Zhou, Qingcao Li

**Affiliations:** ^1^Department of Hospital Infection-Control, The Affiliated Li Huili Hospital, Ningbo University, Ningbo, China; ^2^Department of Clinical Laboratory, Ninghai County Chengguan Hospital, Ningbo, China; ^3^Department of Clinical Laboratory, The Affiliated Li Huili Hospital, Ningbo University, Ningbo, China; ^4^Department of Clinical Infectious Disease, The Affiliated Li Huili Hospital, Ningbo University, Ningbo, China

**Keywords:** CRKP, carbapenemase, ceftazidime-avibactam, aztreonam, combined drug sensitivity

## Abstract

**Objective:**

This study aims to investigate the synergistic effects and biofilm inhibition mechanisms of ceftazidime-avibactam (CZA) combined with aztreonam (ATM) against carbapenem-resistant *Klebsiella pneumoni*a (CRKP) commonly found in the local clinical setting, providing new insights for clinical anti-infective strategies.

**Methods:**

We selected a total of 150 non-duplicate clinical isolates of CRKP from multiple hospitals in Ningbo. Common carbapenemase genes were detected using PCR. Broth microdilution and time-kill assays were used to evaluate the *in vitro* synergistic effects of CZA and ATM, both individually and in combination, on CRKP isolates with different enzyme types, and the fractional inhibitory concentration index (FICI) was calculated. The crystal violet staining method and bacterial cell permeability assay were employed to assess the impact of CZA, ATM, and their combination on the cell structure and biofilm formation capacity of CRKP. Real-time quantitative PCR (qRT-PCR) was used to measure the expression levels of biofilm-related genes (*Luxs*, *mrkA*, *wbbM*, *pgaA*, and *wzm*) in CRKP under treatment with CZA, ATM, or their combination.

**Results:**

The comparison of synergistic indices for different enzyme-type CRKP strains with CZA and ATM combination therapy showed a statistically significant difference (*p* < 0.01). The time-kill assay indicated that the time-kill curves for strains carrying *blaKPC-2* and *blaNDM-1* resistance genes were similar between the monotherapy and combination therapy groups, while the CZA + ATM combination therapy group showed a significant decrease in bacterial concentration after 4–8 h of cultivation compared to the CZA and ATM monotherapy groups. The crystal violet staining and bacterial cell permeability assays demonstrated that the CZA + ATM combination significantly reduced biofilm formation and increased cellular structure disruption in CRKP. The qRT-PCR results showed that CZA combined with ATM notably decreased the expression levels of biofilm-related genes *Luxs*, *mrkA*, *wbbM*, *pgaA*, and *wzm* in CRKP.

**Conclusion:**

The combination of ATM and CZA shows a strong synergistic antibacterial effect against CRKP strains with various enzyme types, with particularly notable synergy in strains carrying the *blaKPC-2* resistance gene. Additionally, this combination significantly disrupts the cellular structure of CRKP and inhibits biofilm formation.

## 1 Introduction

**Figure 1:**
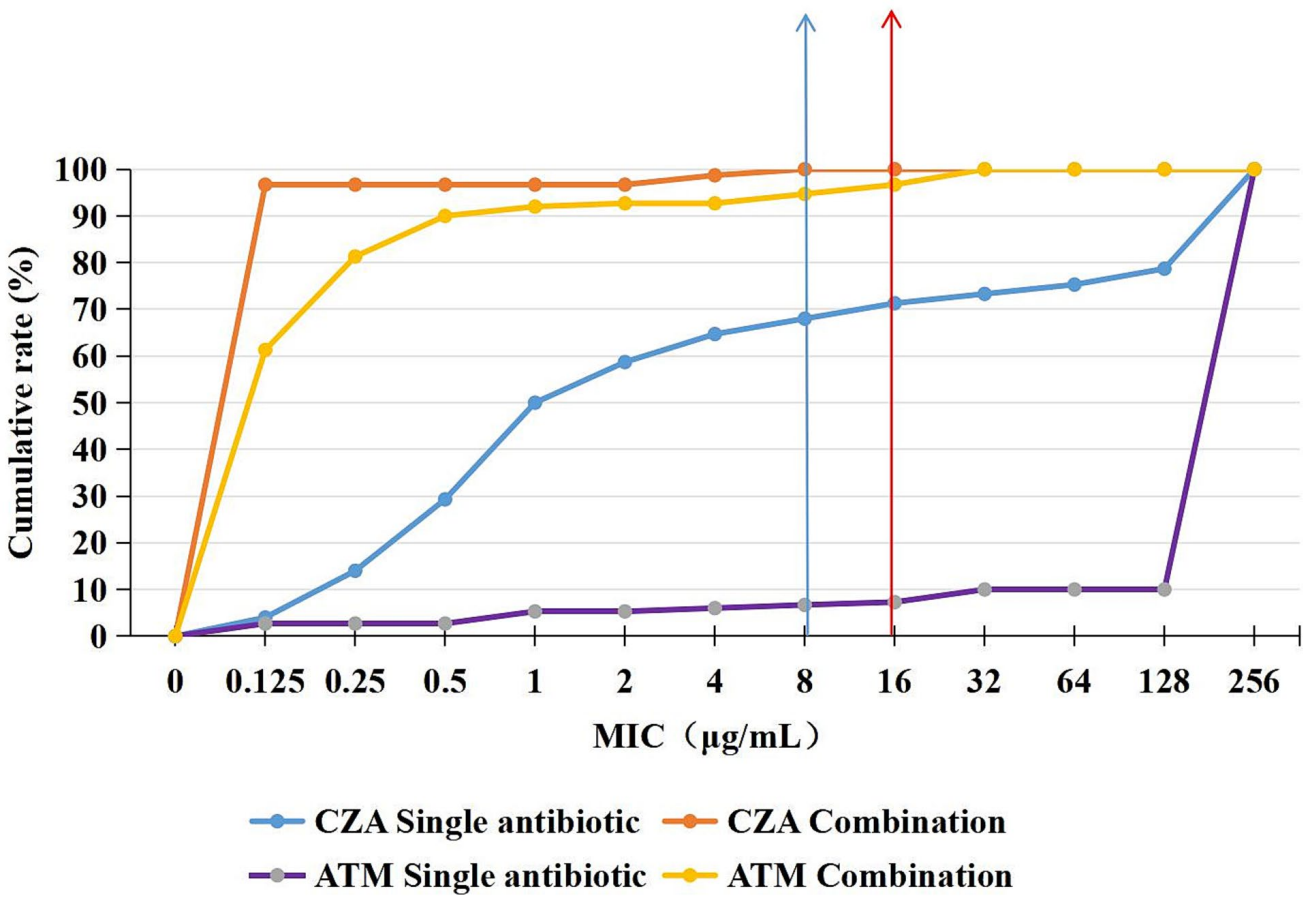
Cumulative rate curves of CZA and ATM monotherapy and combination therapy. After the combination of CZA and ATM, the sensitivity rate significantly increased.

*Klebsiella pneumoniae* (KP) is an opportunistic pathogen widely recognized as a major cause of various severe infections, including liver abscesses, urinary tract infections, and surgical site infections. More concerningly (Vachvanichsanong et al., 2020; Zhang et al., 2022), KP is also a common multidrug-resistant organism in hospital settings, posing significant challenges to treatment (Bassetti et al., 2018). Carbapenem antibiotics have a broad antibacterial spectrum and excellent antibacterial activity, particularly showing outstanding effectiveness against *Enterobacteriaceae* that produce extended-spectrum *β*-lactamases and cephalosporinases. In recent years, with the extensive and even excessive use of these drugs, the detection rate of CRKP has continued to rise, leaving very few treatment options available. At the same time, the mortality rate associated with infections caused by CRKP has been increasing year by year. This phenomenon poses a significant challenge to the clinical treatment of KP infections (Gonçalves Barbosa et al., 2022). In recent years, expert consensus domestically (Guan et al., 2016) and internationally on the treatment of multidrug-resistant and even pan-drug-resistant Enterobacteriaceae has emphasized that the treatment of CRKP infections should follow the principles of “early, accurate, adequate, and combined” therapy. Combination regimens based on tigecycline, polymyxin, and CZA are recommended. However, the clinical application of tigecycline and polymyxin is limited due to issues like low blood drug concentrations, heterogeneous resistance, and side effects, such as liver and kidney toxicity (Genteluci et al., 2020; Ballı et al., 2024; Turan Gökçe et al., 2024). In contrast, CZA shows high sensitivity in treating CRKP, with good prognosis and there are few adverse reactions during the anti-infective process (Sanz Herrero, 2022). However, CZA is ineffective against strains producing metallo-*β*-lactamases (including types such as *blaNDM*, *blaVIM*, and *blaIMP*), which limits its range of use to some extent. Therefore, based on aztreonam’s inhibitory effect on metallo-β-lactamases, some researchers have proposed a combination therapy of CZA and ATM (Sempere et al., 2022). In previous clinical studies, our research team also observed the *in vitro* synergistic effect of CZA and ATM (Figure 1). Currently, there are relatively few studies reporting on the combined use of these two drugs. We will conduct an in-depth analysis of the carbapenemase types present in CRKP strains in this region and clarify the differences in drug susceptibility and synergy indices of CZA combined with ATM against CRKP with various enzyme types. Additionally, we will further investigate the effects of CZA combined with ATM on the structural disruption of CRKP cells and the inhibition of biofilm formation, providing a strong basis for clinical treatment.

## 2 Materials and methods

### 2.1 Bacterial strains and specimen source

This study collected 150 strains of CRKP from multiple centers in Ningbo as research samples (excluding duplicate isolates). These strains were primarily sourced from various types of clinical samples, including sputum, blood, drainage fluid, wound secretions, and midstream urine. All isolates were identified by matrix-assisted laser desorption/ionization time-of-flight mass spectrometry (Zhongyuan, China). This study was approved by the Ethics Committee of Ningbo Medical Centre Lihuili Hospital, Ningbo University (KY2023SL347-01). *Escherichia coli* ATCC 25922 and *Pseudomonas aeruginosa* ATCC27853 served as the quality control strain for strain identification and antimicrobial susceptibility testing, all quality control strains were purchased from the National Centre for Clinical Laboratories, Ministry of Health.

### 2.2 Preparation of DNA templates and detection of carbapenemase resistance genes

DNA template was extracted using a kit (Shenggong, China). Premix TaqTM DNA Polymerase (TaKaRa, Japan) was used for PCR amplification of carbapenemase resistance genes. The relevant primers sequences (*blaKPC-2, blaIMP-1, blaIMP-2, blaNDM-1, blaVIM, blaOXA-48 和 blaOXA-23*) are listed in Table 1. The PCR mixture consisted of a total volume of 25 μL, comprising 1 μL of genomic DNA template, 1 μL of each primer, 12.5 μL of Premix-rTaq PCR solution (manufactured by TaKaRa, Japan), and 9.5 μL of distilled water. The PCR procedure was conducted utilizing an ABI Veriti Thermal Cycler (Applied Biosystems, Singapore). The template was initially subjected to denaturation at a temperature of 94°C for a duration of 5 min. This was followed by 30 cycles consisting of denaturation at 94°C for 45 s, annealing at 55°C for 45 s, and extension at 72°C for 1 min. A final extension step was performed at 72°C for 10 min. The PCR products were subsequently confirmed through electrophoresis and sequencing.

**Table 1:** The primers and their sequences for integron screening.

Primer	Primer sequence (5′-3′)	Tm (°C)	Reference
*KPC-2-F*	ATGTCACTGTATCGCCGTCT	55	Liu et al. (2021)
*KPC-2-R*	TTTTCAGAGCCTTACTGCCC		
*IMP-1-F*	CATGGTTTGGTGGTTCTTGT	55	Liu et al. (2021)
*IMP-1-R*	GTAMGTTTCAAGAGTGATGC		
*IMP-2-F*	GGCAGTCGCCCTAAAACAAA	55	Martirosov and Lodise (2016)
*IMP-2-R*	TAGTTACTTGGCTGTGATGG		
*VIM-F*	GTTTGGTCGCATATCGCAAC	55	Liu et al. (2021)
*VIM-R*	CTACTCGGCGACTGAGCGAT		
*NDM-1-F*	CAGCACACTTCCTATCTC	55	Liu et al. (2021)
*NDM-1-R*	CCGCAACCATCCCCTCTT		
*OXA-48-F*	TTGGTGGCATCGATTATCGG	55	Liu et al. (2021)
*OXA-48-R*	GAGCACTTCTTTTGTGATGGC		
*OXA-23-F*	GATCGGATTGGAGAACCAGA	55	El-Badawy et al. (2020)
*OXA-23-R*	ATTTCTGACCGCATTTCCAT		
*luxS-F*	AGTGATGCCGGAACGCGG	60	Vuotto et al. (2017)
*luxS-R*	CGGTGTACCAATCAGGCTC		
*mrkA-F*	ACGTCTCTAACTGCCAGGC	60	Vuotto et al. (2017)
*mrkA-R*	TAGCCCTGTTGTTTGCTGGT		
*wbbM-F*	ATGCGGGTGAGAACAAACCA	62	Vuotto et al. (2017)
*wbbM-R*	AGCCGCTAACGACATCTGAC		
*pgaA-F*	GCAGACGCTCTCCTATGTC	60	Vuotto et al. (2017)
*pgaA-R*	GCCGAGAGCAGGGGAATC		
*rpoB-F*	AGGCGAATCCAGCTTGTTCAGC	62	Vuotto et al. (2017)
*rpoB-R*	TGACGTTGCATGTTCGCACCCATCA		

### 2.3 Drug susceptibility testing of CZA and ATM alone and in combination

To determine the combined effect of CZA and ATM, we used the broth microdilution checkerboard method, setting the concentration gradient range of ceftazidime (Glpbio, America) -avibactam (Glpbio, America) and ATM (Glpbio, America) based on the MIC of CZA, with a range of 0.125–256 μg/mL. Briefly, CZA was used as drug A and ATM were prepared used as drug B. The two drugs were diluted in rows and columns, respectively, using twofold serial dilutions to obtain dilution results for different concentration combinations. The tested strain was inoculated, with quality control as a reference. After incubation at 37°C overnight, the MIC values for the combination were recorded. The synergistic effect of CZA combined with ATM was investigated using the FICI. FICI = FIC_A_ + FIC_B_; FIC_A_ = MIC_A_ in combination/MIC_A_ alone; FIC_B_ = MIC_B_ in combination/MIC_B_ alone, and interpretation criteria were based on reference (Lin et al., 2021). CZA and ATM were evaluated according to the 2023 Clinical and Laboratory Standards Institute (CLSI) standards.

### 2.4 Analysis of growth ability of experimental strains

We selected 8 strains of CRKP carrying *blaKPC-2* resistance gene and *blaNDM-1* resistance gene based on the MICs of CZA and ATM against CRKP respectively, we investigated the bactericidal effect of CZA combined with ATM at 0.5, 1, and 2 MICs using time-kill curves. Two to three colonies were inoculated into 2 mL of MH broth (Shenggong, China) and incubated at 35°C for 18–20 h overnight. The bacterial suspension was adjusted to a 0.5 McFarland standard, then diluted 10,000-fold with MH broth (T-2 count approximately 10^4 CFU/mL), and incubated for 2 h on a shaking incubator (T + 0, approximately 10^5 CFU/mL). The bacterial suspension containing 1 × 10^5 CFU/mL was then mixed with either single or combined antimicrobial agents, and incubated overnight at 35°C with continuous shaking. A control group with no antibiotics was prepared in the same manner. At 0, 2, 4, 6, 8, and 24 h, 10-fold serial dilutions of the broth samples were made, and 100 μL of each diluted sample was plated onto MH agar plates in triplicate. After overnight incubation at 35°C, colonies were counted and the average colony count was recorded. The time-kill curves for each strain were plotted with the bacterial count (CFU/mL) on the y-axis and time (hours) on the x-axis.

### 2.5 Detection of biofilm formation ability

For the selected strains mentioned above, we stained the biofilm with crystal violet. After inoculating the strains for 18 h, they were diluted 1:100 in fresh MH broth. Then, 200 μL of the MH broth was transferred to a 96-well microtiter plate (flat-bottomed, with lid, sterile), and experimental strains (1 × 10^6 CFU/mL) were inoculated into 10 mL of LB broth containing CZA (½ MIC), ATM (½ MIC), CZA (½ MIC) + ATM (½ MIC), and LB broth without antibiotics as a control. The plate was incubated at 37°C for 48 h. After incubation, planktonic bacteria were discarded, and the wells were washed twice with saline and air-dried. Next, 200 μL of methanol was added to fix the biofilm for 15 min, after which the methanol was removed. Following this, 200 μL of crystal violet solution (Shenggong, China) was added to stain the biofilm for 15 min, and the dye solution was discarded. The wells were washed twice with saline and air-dried. Then, 200 μL of absolute ethanol was added to each well, and the plate was left to stand for 10 min. Finally, the absorbance at 595 nm (A595) was measured, the average of three replicates was calculated, and the process was repeated three times.

### 2.6 Permeability test of bacterial cells

For the selected strains mentioned above, we evaluated the permeability of bacterial cells using alkaline phosphatase (ALP). Experimental strains (1 × 10^6 CFU/mL) were inoculated into 10 mL of LB broth containing CZA (½ MIC), ATM (½ MIC), CZA (½ MIC) + ATM (½ MIC), and LB broth without antibiotics as a control. The cultures were then incubated in a shaking incubator at 37°C and 180 rpm for 24 h. After incubation, extract ALP according to the ALP kit method (Shenggong, China) (ALP is commonly present inside bacterial cells, and an increase in ALP leakage can indicate cell structure damage). Generally, ALP activity is measured using p-nitrophenyl phosphate (pNPP) as the phosphatase substrate, which turns yellow upon dephosphorylation by ALP (λmax = 510 nm). ALP activity is calculated by measuring the absorbance at 510 nm, the average of three replicates was calculated, and the process was repeated three times.

### 2.7 Detection of expression of biofilm-related genes

We selected CRKP strains carrying different resistance genes (as described above) for qRT-PCR analysis to examine the expression levels of biofilm-related genes (*LuxS*, *mrkA*, *wbbM*, *pgaA*, and *wzm*) under conditions of CZA and ATM monotherapy and combination therapy. Primers were synthesized according to the literature (Table 1). To ensure the accuracy of gene expression results, we first assessed cell viability under each treatment condition using the colony-forming unit (CFU) assay. Briefly, bacterial cultures were treated with CZA and ATM at their respective MIC values (alone and in combination) for 6 h. After treatment, serial dilutions of the bacterial suspensions were plated on Mueller-Hinton agar plates and incubated at 37°C for 24 h. The CFU counts were then recorded to determine the viability of CRKP under each treatment condition. Only samples with comparable cell viability across treatment groups were used for subsequent qRT-PCR analysis. Simultaneously, we established both an untreated control group (CRKP strains cultured without antibiotics) and a solvent-only control group (CRKP strains treated with the solvent used to dissolve CZA and ATM) to exclude the influence of non-specific factors on the experimental results. Lastly, we conducted statistical analyses on the differences in gene expression among various treatment groups to verify whether these differences were statistically significant. For qRT-PCR, total RNA was extracted from bacterial cells using the TRIzol reagent (Invitrogen, United States) according to the manufacturer’s instructions. RNA quality and concentration were measured using a NanoDrop spectrophotometer (Thermo Fisher Scientific, United States). cDNA was synthesized using the PrimeScript RT Reagent Kit (Takara, Japan). qRT-PCR was performed using the SYBR Green Master Mix (Shenggong, China) on a QuantStudio 5 Real-Time PCR System (Applied Biosystems, United States). The relative expression levels of target genes were calculated using the 2^−ΔΔCt method, with rpoB as the reference gene. Each experiment was performed in triplicate to ensure reproducibility.

### 2.8 Statistical analysis

The clinical strain data were analyzed in depth using WHONET 5.6 software and SPSS 25.0 statistical software. The statistical results indicated that a *p*-value of less than 0.05 was considered statistically significant.

## 3 Results

### 3.1 The results for CRKP carbapenemase type by PCR

Among the 150 CRKP strains, those carrying the *blaKPC-2* resistance gene were predominant, totaling 92 strains and accounting for 61.3% of the total strains. Additionally, 22 strains carried the *blaNDM-1* resistance gene, representing 14.7% (Table 2). Unfortunately, no strains carrying the *blaVIM-1*, *blaVIM-2*, or *blaIMP-2* resistance genes were detected.

**Table 2:** Distribution of carbapenemase resistance genes carried by CRKP.

Resistance mechanism	Genotype	Number	Proportion
Carbapenemase	*KPC-2*	92	61.3
	*NDM-1*	22	14.7
	*NDM-2*	0	0.0
	*VIM*	0	0.0
	*IMP-1*	12	8.0
	*IMP-2*	0	0.0
	*OXA-23*	9	6.0
	*OXA-48*	2	1.3
	*KPC-2 + NDM-1*	2	1.3
	*KPC-2 + IMP-1*	3	2.0
	Other	8	5.4
	Total	150	100

### 3.2 The results of sensitivity of CZA and ATM alone and in combination to CRKP *in vitro* by broth dilution checkerboard method

The broth microdilution checkerboard method was used to determine the susceptibility rates of CZA monotherapy and combination therapy against CRKP. The results showed susceptibility rates of 68 and 100%, respectively. Similarly, the susceptibility rates for ATM monotherapy and combination therapy were 6 and 92.7%, indicating a significant increase in susceptibility after combination therapy (Figure 1). Additionally, when comparing the MIC50 and MIC90 values of CZA and ATM monotherapy versus combination therapy, we found significant reductions in both values after combination therapy (*p* < 0.05). The specific results are shown in Table 3.

**Table 3:** The MIC distribution, accumulation rate, MIC50, and MIC90 of CZA and ATM single and in combination were measured by broth microdilution method.

MIC (μg/mL)	CZA				ATM			
	Single antibiotic		Combination		Single antibiotic		Combination	
	Number	Cumulative rate (%)	Number	Cumulative rate (%)	Number	Cumulative rate (%)	Number	Cumulative rate (%)
0.125	6	4.0	145	96.7	4	2.7	92	61.3
0.25	21	14.0	145	96.7	4	2.7	122	81.3
0.5	44	29.3	145	96.7	4	2.7	135	90.0
1	75	50.0	145	96.7	8	5.3	138	92.0
2	88	58.7	145	96.7	8	5.3	139	92.7
4	97	64.7	148	98.7	9	6.0	139	92.7
8	102	68.0	150	100.0	10	6.7	142	94.7
16	107	71.3	150	100.0	11	7.3	145	96.7
32	110	73.3	150	100.0	15	10.0	150	100
64	113	75.3	150	100.0	15	10.0	150	100.0
128	118	78.7	150	100.0	15	10.0	150	100.0
256	150	100.0	150	100.0	150	100.0	150	100.0
MIC range	0.125 ~ 256		0.125 ~ 16		0.125 ~ 256		0.125 ~ 64	
MIC_50_	1		0.125		256		0.125	
MIC_90_	256		0.125		256		0.5	
Z1	−10.280							
P1	<0.01							
Z2	−10.683							
P2	<0.01							

Z1 and P1 are comparisons between CZA single and combination therapy, and Z1 and P2 are comparisons between ATM single and combination therapy.

### 3.3 The distribution of antibacterial synergistic index of CZA and ATM combined against CRKP producing different enzyme types

The synergy index determined by the broth microdilution checkerboard method showed that 90.7% of the FICI were ≤ 0.5, 0.7% were between 0.5 and 1, 7.3% were between 1 and 2, and 1.3% were > 2. Furthermore, when CZA and ATM were used in combination, the proportion of FICI ≤0.5 for CRKP producing A, B, and D class enzymes was 93.5, 85.3, and 72.7%, respectively. Comparison of the synergy index for different enzyme types of CRKP after combination therapy revealed statistically significant differences (*p* < 0.01). The specific results are shown in Table 4.

**Table 4:** Distribution of synergy index of combined drug therapy on different enzyme types of CRKP [*N* (%)].

FICI	Class A enzymes	Class B enzymes	Class D enzymes	*p*
≤0.5	86 (93.5)	29 (85.3)	8 (72.7)	<0.01
0.5 < FICI ≤ 1	0 (0)	0 (0)	1 (9.1)	
1 < FICI ≤ 2	5 (5.4)	4 (11.8)	1 (18.2)	
FICI > 2	1 (1.1)	1 (2.9)	0 (0)	
Total	92	34	11	

FICI, fractional inhibitory concentration index.

### 3.4 Results of growth ability analysis of CRKP under single-drug and combined-drug therapy

**Figure 2:**
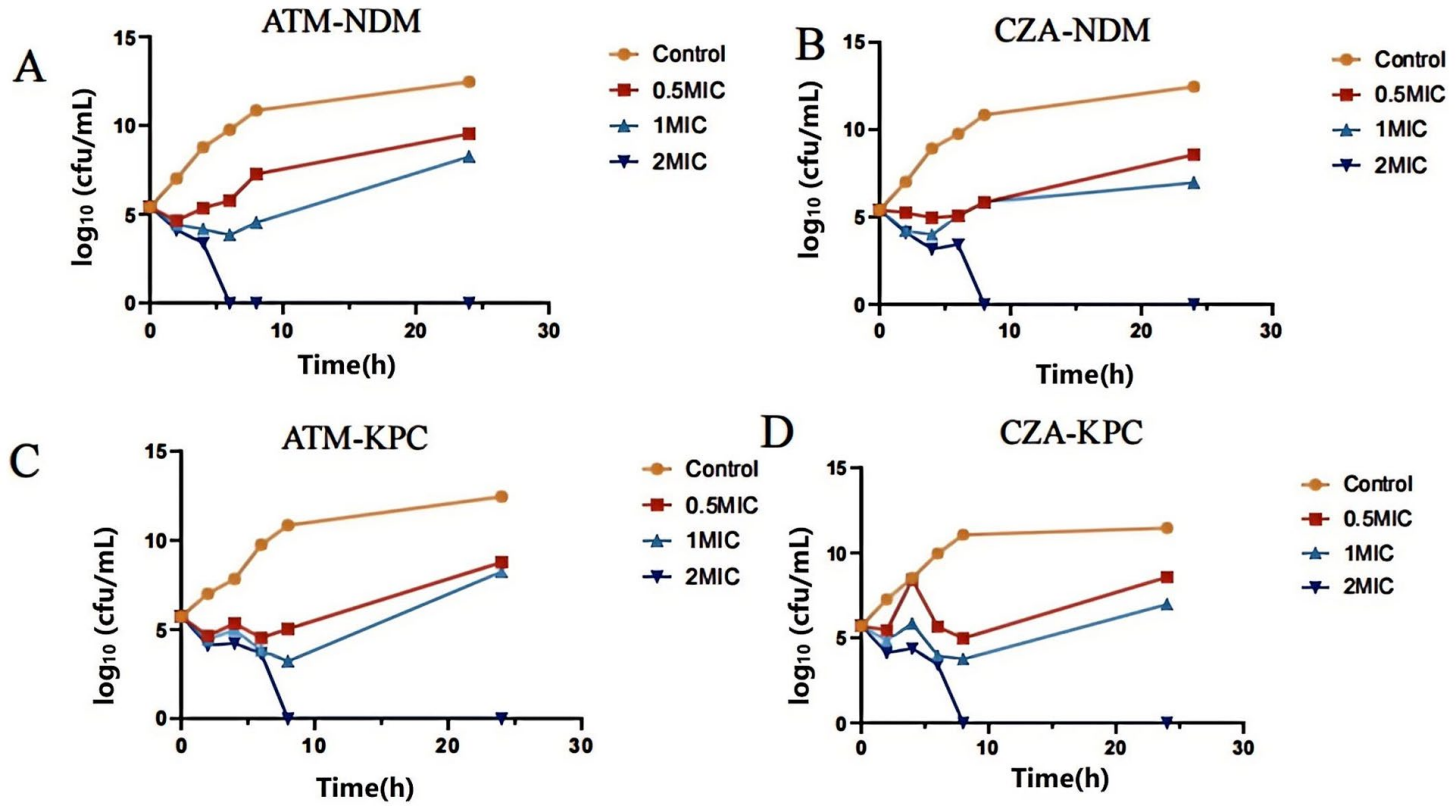
**(A)** Time sterilization curves of CRKP carrying *blaNDM* resistance gene by different concentrations of ATM. **(B)** Time sterilization curves of CRKP carrying *blaNDM* resistance gene with different concentrations of CZA. **(C)** Time sterilization curves of CRKP carrying *blaKPC* resistance gene by different concentrations of ATM. **(D)** Time sterilization curves of CRKP carrying *blaKPC* resistance gene with different concentrations of CZA.

**Figure 3:**
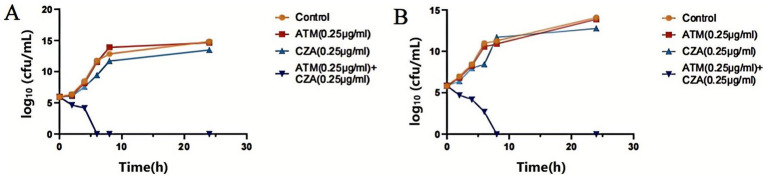
**(A)** Time sterilization curves of ATM, CZA monotherapy, and combination on CRKP carrying *blaNDM* resistance gene. **(B)** Time sterilization curves of ATM, CZA monotherapy, and combination on CRKP carrying *blaKPC* resistance gene.

The time-kill curve demonstrated that CRKP carrying the *blaNDM-1* resistance gene and CRKP carrying the *blaKPC-2* resistance gene showed a rebound in bacterial growth after 6–8 h of incubation at 0.5 and 1 MIC concentrations of ATM and CZA, respectively. However, at 2 MIC of ATM and CZA, bacterial counts steadily decreased within 2 h of incubation, and no colony formation was observed after 6–8 h, indicating significant bactericidal effects against CRKP (Figure 2). When ATM and CZA were used in combination, the bacterial concentrations of CRKP carrying the *blaNDM-1* resistance gene and CRKP carrying the *blaKPC-2* resistance gene both decrease rapidly after 4–6 h of culture, indicating that the combined treatment has a strong bactericidal effect on strains with different enzyme types. After 8 h of combination therapy, no bacterial growth was detected (Figure 3).

### 3.5 Results of biofilm formation ability test

**Figure 4:**
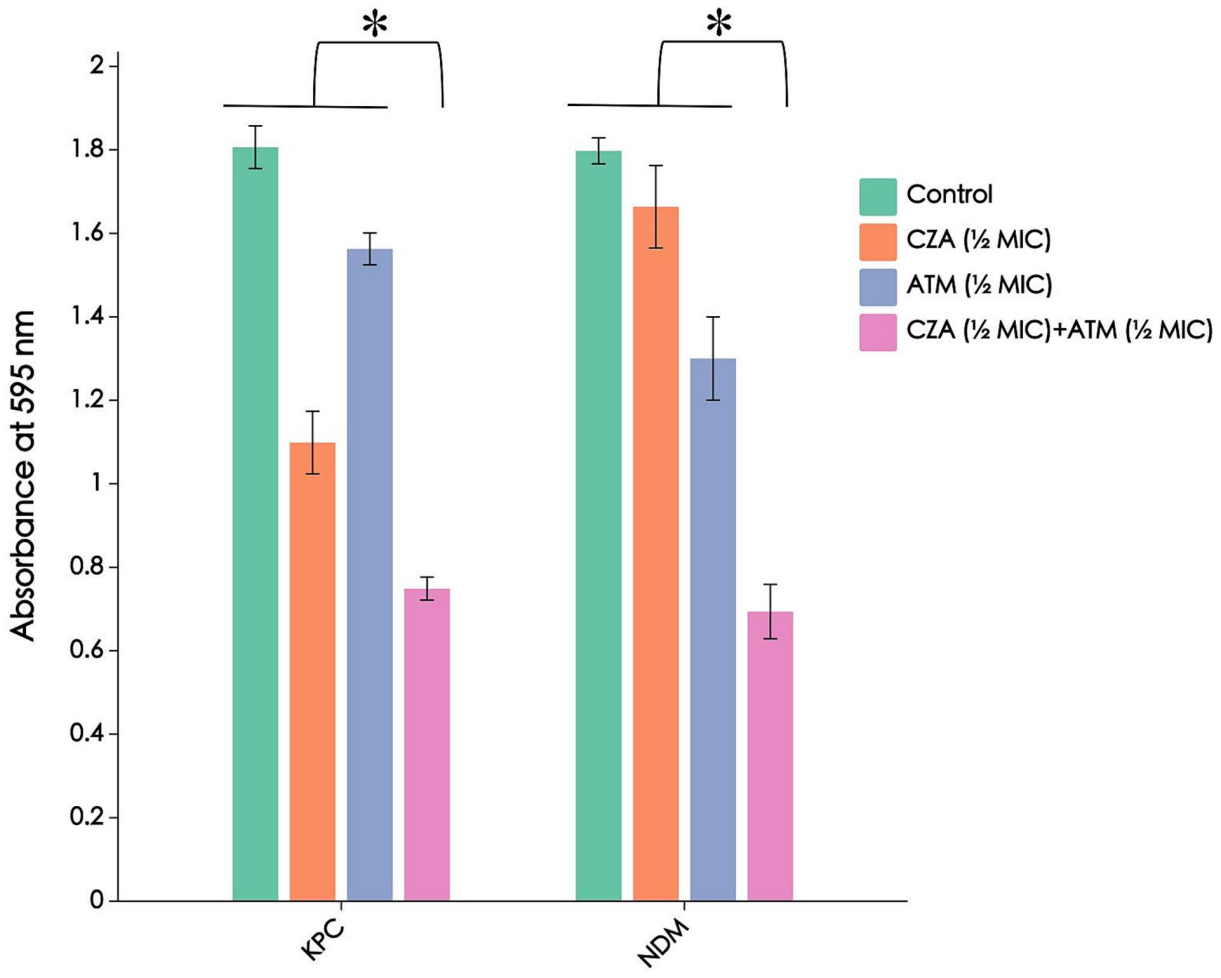
The absorbance of different enzyme types of CRKP biofilms stained with crystal violet after ATM, CZA monotherapy, and combination treatment. The combined use of ATM and CZA inhibits the formation of biofilms carrying *blaNDM* and *blaKPC* resistance genes in CRKP. *p < 0.01, determined by the student’s *t*-test.

In the absence of any antibiotics, CRKP strains carrying *blaNDM* and *blaKPC* resistance genes both exhibited strong biofilm formation. When single or combined antibiotics were added, the biofilm formation of all strains was inhibited compared to the untreated group, with a more significant reduction in absorbance observed in the combination treatment group (*p* < 0.01). Furthermore, when comparing CRKP strains carrying *blaNDM* and *blaKPC* resistance genes, it was found that CRKP carrying *blaKPC* showed more pronounced biofilm inhibition after treatment with CZA than CRKP carrying *blaNDM*, while the effect of ATM was the opposite. The results are shown in Figure 4.

### 3.6 Results of bacterial cell permeability test

**Figure 5:**
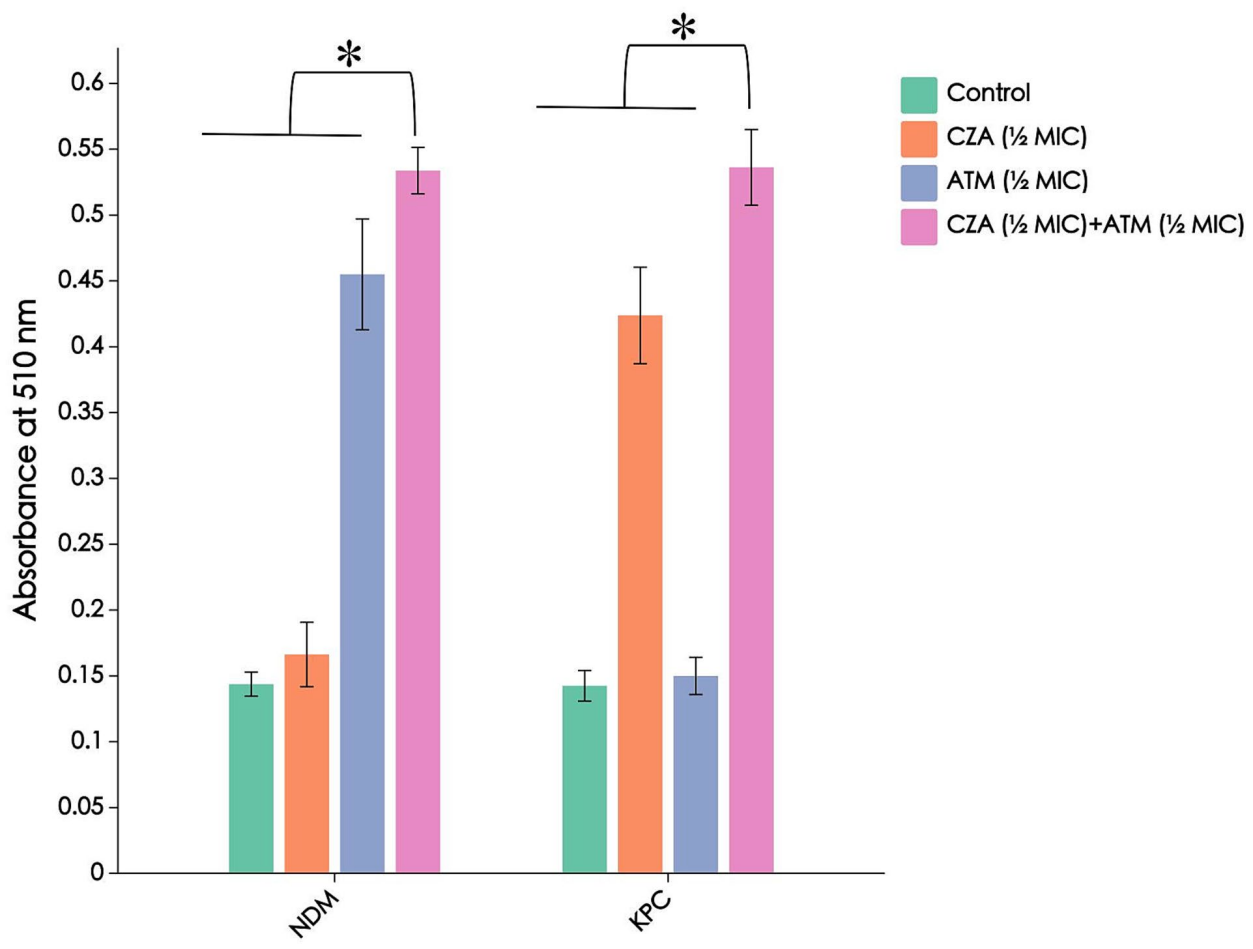
Combination of CZA and ATM contributes to the damage of bacterial cell membranes and the leakage of bacterial ALPs. The absorbance at 510 nm integrity by detecting ALP. The chart indicates that the combination of two drugs acts on CRKP carrying *blaNDM* and *blaKPC* resistance genes, an increase for alkaline phosphatase in the medium after exposure to the specified compound. *p < 0.01, determined by the student’s *t*-test.

Without the addition of antibiotics, the CRKP cell permeability was low, and the ALP content in the supernatant was minimal, resulting in a weak absorbance after the reaction. With the addition of single or combination antibiotics, the absorbance significantly increased, with the ATM + CZA combination group showing a notably higher absorbance compared to the monotherapy groups (*p* < 0.05). Furthermore, when comparing CRKP strains carrying *blaNDM* and *blaKPC* resistance genes, it was found that CRKP carrying *blaKPC* showed a notably higher absorbance after treatment with CZA than CRKP carrying *blaNDM*, while the effect of ATM was the opposite. The results are shown in Figure 5.

### 3.7 Detection results of biofilm-related gene expression

**Figure 6:**
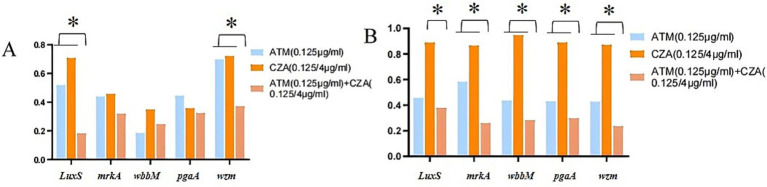
**(A)** The expression levels of biofilm formation related genes in CRKP carrying *blaKPC* resistance gene by ATM, CZA monotherapy, and combination. **(B)** The expression levels of biofilm formation related genes in CRKP carrying *blaNDM* resistance gene by ATM, CZA monotherapy, and combination, *p < 0.01, determined by the student’s *t*-test.

The analysis of gene expression levels related to biofilm formation in CRKP revealed that, compared to the untreated group, the expression levels of genes encoding biofilm-associated proteins (*LuxS*, *mrkA*, *wbbM*, *pgaA*, and *wzm*) were reduced in both the single antibiotic group and the combined antibiotic group. Notably, in CRKP carrying the *blaKPC* resistance gene, the expression levels of the *luxS* and *wzm* genes were significantly lower after treatment with the ATM + CZA combination compared to treatment with a single antibiotic alone (*p* < 0.01). Similarly, in CRKP carrying the *blaNDM* resistance gene, the expression levels after treatment with the ATM + CZA combination were approximately 3 to 5 times lower than after treatment with CZA alone (*p* < 0.01). The results are shown in Figure 6.

Based on the experimental data and related results presented above, including the distribution of carbapenemase types among CRKP strains, the susceptibility rates of CZA and ATM alone and in combination, the synergistic effects of combination therapy, the time-kill curve analysis, biofilm formation inhibition, bacterial cell permeability changes, and the expression levels of biofilm-related genes, we now proceed to the following discussion. These findings collectively provide a comprehensive understanding of the efficacy and mechanisms of CZA and ATM combination therapy against CRKP, as well as insights into the potential clinical applications of this treatment strategy.

## 4 Discussion

*Klebsiella pneumoniae*, a species of *Enterobacteriaceae*, can cause diseases under specific conditions. In recent years, with the widespread use of carbapenem antibiotics in clinical settings, the detection rate of CRKP has been increasing annually. This bacterium is often referred to as a “superbug” due to its resistance to more than 70 types of antibiotics. Currently, treatment options for CRKP are relatively limited, primarily consisting of drugs such as tigecycline, polymyxins, fosfomycin, and aminoglycosides (especially gentamicin and amikacin) (Livermore et al., 2011). However, some of these drugs are expensive and are associated with significant side effects. More seriously, the use of these drugs alone can lead to a gradual increase in pathogen resistance, which makes treatment more challenging and often results in the failure of conventional treatment regimens, ultimately affecting treatment efficacy and success rates. CZA, as a novel *β*-lactamase inhibitor, is known for its high sensitivity and low adverse effects. However, its efficacy against strains producing metallo-β-lactamases is not ideal, which limits its range of use (Wang et al., 2020). Although there have been reports of combination therapy with CZA and ATM, studies on their synergistic mechanisms and differences between CRKP strains with varying enzyme types are relatively rare. This study aims to analyze the carbapenemase profile of CRKP strains in our region, investigate the differences in drug sensitivity and synergy index between CZA and ATM combination therapy for different enzyme types, and further elucidate their effects on CRKP cell structure disruption and biofilm inhibition. The goal is to provide a solid basis for clinical treatment strategies.

In clinically isolated carbapenemase-producing strains in China, strains producing *blaKPC* and *blaNDM*-type enzymes are the most prevalent, while a smaller number of strains produce *balOXA-48*, *blaIMP*, and *blaVIM*-type carbapenemases (Munoz-Price et al., 2013). In this study, among the 150 CRKP strains, those carrying *blaKPC-2* and *blaNDM-1* resistance genes were dominant (Table 2). Interestingly, we also identified strains carrying both *blaKPC-2* and *blaNDM-1* resistance genes, as well as strains carrying *blaKPC-2* and *blaIMP-1* resistance genes. But no strains carrying the *blaVIM-1*, *blaVIM-2*, or *blaIMP-2* resistance genes were identified. This result may be related to the following factors: regional epidemiological characteristics, limitations in sample sources, and gene transmission mechanisms. The prevalence of *blaVIM* and *blaIMP* genes varies significantly across different regions. For example, *blaVIM*-type carbapenemases are more commonly reported in the Mediterranean region and some parts of Asia (Peirano et al., 2012), while *blaIMP*-type enzymes are frequently documented in East Asia (e.g., Japan) (Matsumura et al., 2017). The strains involved in this study may originate from regions where *blaVIM* and *blaIMP* genes are not prevalent, resulting in a lower detection rate. Additionally, the samples in this study were primarily clinical isolates from a specific region, which may not fully represent the diversity of strains in other areas or specific environments. Furthermore, *blaVIM* and *blaIMP* genes are often located on mobile genetic elements (e.g., plasmids or integrons) (Izdebski et al., 2023), and their transmission may be influenced by factors such as host adaptability and antibiotic selection pressure. In the environment of this study, the spread of these genes may be restricted. Although *blaVIM* and *blaIMP* genes were not detected in this study, their absence does not rule out the possibility of their presence in other regions or specific clinical settings. In future research, we plan to expand the sample size and incorporate molecular epidemiological analysis to further investigate the distribution patterns and transmission mechanisms of these resistance genes. Furthermore, strains producing different types of enzymes exhibit significant differences in resistance characteristics. The sensitivity of CRKP carrying different carbapenemase genes to antimicrobial drugs also varies, thereby affecting drug selection (Munoz-Price et al., 2013). Therefore, we selected CRKP strains carrying different resistance genes to conduct various experiments, investigating whether the combination of ATM and CZA exhibits the same synergistic and antibacterial effects against different enzyme types of CRKP.

The susceptibility rates and MIC values of CZA and ATM against CRKP, as determined by the microdilution broth method, are summarized in Table 3. Our results showed that the susceptibility rates of CZA and ATM monotherapy were 64.7 and 6%, respectively, indicating relatively low efficacy against CRKP when used alone. Further analysis revealed that CZA demonstrated strong resistance against CRKP strains producing metallo-*β*-lactamases, while ATM, although showing some inhibitory effect against metallo-β-lactamase-producing strains, still had a very high overall resistance rate against CRKP. The resistance of CRKP to ATM and CZA as monotherapy can be attributed to several mechanisms. Firstly, CRKP often produces β-lactamases, including carbapenemases (e.g., KPC, NDM) and extended-spectrum β-lactamases (ESBLs), which hydrolyze β-lactam antibiotics, rendering them ineffective (Bush and Bradford, 2020). Additionally, efflux pumps may contribute to resistance by actively expelling antibiotics from bacterial cells, reducing intracellular drug concentrations (Amereh et al., 2023). Moreover, genetic mutations of KPC variants and penicillin-binding proteins (PBPs) may further diminish the binding affinity of β-lactam drugs, limiting their bactericidal activity (Cavallini et al., 2021). The significant reduction in MIC values for CZA and ATM in combination compared to their use alone, along with the statistically significant differences observed (*p* < 0.01), underscores the enhanced efficacy of the combination therapy. To further elucidate the synergistic effects, we performed FIC analysis, which revealed synergistic effects (FICI ≤0.5) in 136 out of 150 CRKP strains (90.7%) and additive effects in only one strain. This high proportion of synergistic interactions indicates that the combination of CZA and ATM effectively compensates for the limitations of monotherapy, providing a robust bactericidal effect against the majority of CRKP strains. Importantly, the clinical relevance of these findings is highlighted by the fact that FICI≤0.5 is widely recognized as a strong indicator of synergistic activity, suggesting that this combination could significantly improve treatment outcomes in patients with CRKP infections. Furthermore, we analyzed the synergistic index of the two drugs against CRKP producing different classes of enzymes. The results showed the highest synergy rates against CRKP producing class A enzymes (93.5%), followed by class B (85.3%) and class D (72.7%) enzymes, with statistically significant differences among groups (*p* < 0.01) (Table 4). This variation in synergy rates may be attributed to differences in the mechanisms of resistance mediated by these enzymes. For instance, the avibactam component of CZA is highly effective against class A enzymes (e.g., KPC), while ATM exhibits stability against class B enzymes (e.g., NDM). The combination thus overcomes the individual limitations of each drug, providing a broad-spectrum therapeutic option for CRKP infections regardless of the enzyme type. These findings not only validate the synergistic potential of CZA and ATM but also provide a rationale for tailoring combination therapy based on the resistance profile of CRKP strains, which could have significant implications for clinical practice.

The time-kill curve analysis provided critical insights into the dose-dependent bactericidal effects of ATM and CZA against CRKP. At sub-inhibitory concentrations (0.5 and 1 MIC), a transient reduction in bacterial counts was observed, followed by regrowth after 6–8 h of incubation (Figure 2). This phenomenon suggests that suboptimal drug concentrations may exert selective pressure, potentially promoting the survival of resistant subpopulations. In contrast, at 2 MIC, a rapid and sustained reduction in bacterial counts was achieved, with no regrowth observed beyond 6–8 h. This dose-dependent response highlights the importance of achieving sufficiently high drug concentrations in clinical settings to ensure complete eradication of CRKP and prevent the emergence of resistance. Furthermore, the combination of ATM and CZA demonstrated enhanced bactericidal activity effect on strains with different enzyme types. Compared to monotherapy, with a rapid decline in bacterial counts within 4–6 h and no detectable growth after 8 h (Figure 3). This synergistic effect is likely attributable to the complementary mechanisms of action of the two drugs: ATM resisting metallo-*β*-lactamase to some extent, thereby protecting CZA from hydrolysis and allowing it to retain its bactericidal activity (Lahiri et al., 2013). Meanwhile, CZA inhibits extended-spectrum-β-lactamases and carbapenemases other than metallo-β-lactamases, ensuring ATM remains undegraded. Additionally, the bactericidal effects of CZA’s ceftazidime component and ATM are mediated through binding to PBPs. Together, the combination enhances bactericidal activity and therapeutic effects. The combination not only overcomes individual resistance mechanisms but also minimizes the risk of bacterial regrowth, underscoring its potential as a promising therapeutic strategy for CRKP infections. Clinical reports, both domestic and international, have documented the use of the new combination of CZA and ATM to treat infections caused by *Enterobacterales* producing metallo-β-lactamases (Davido et al., 2017; Marshall et al., 2017). Shaw et al. (2018) also demonstrated that the combination of ATM and CZA is a safe treatment option for severe infections caused by Enterobacterales producing β-lactamases and carbapenemases. The dose-dependent effects observed in our study further support the need for pharmacokinetic/pharmacodynamic (PK/PD) optimization in clinical practice to maximize therapeutic efficacy and minimize resistance development.

Recent studies have shown a strong correlation between bacterial antibiotic resistance and biofilm formation, with biofilms being the cause of approximately 75% of human bacterial infections (Høiby et al., 2010). KP has demonstrated the ability to form biofilms as a means of adapting to adverse environments. In this biofilm state, KP exhibits significant resistance, evades immune system attacks, and poses challenges for complete eradication, complicating clinical anti-infection efforts (Geng et al., 2024). For CRKP with different enzyme types, crystal violet staining showed a significant decrease in absorbance in the combination group compared to the blank and monotherapy groups (Figure 4), indicating a pronounced biofilm inhibitory effect, which aids in treating refractory infections caused by CRKP. Bacterial cell permeability tests revealed that the combination group significantly increased CRKP cell disruption and the leakage of cellular contents (Figure 5), providing structural-level evidence for the bactericidal effect of the CZA + ATM combination. Furthermore, when comparing CRKP strains carrying *blaNDM* and *blaKPC* resistance genes, it was found that CRKP carrying *blaKPC* exhibited more pronounced biofilm inhibition after treatment with CZA than CRKP carrying *blaNDM*, while the effect of ATM was the opposite. This suggests that the efficacy of antibiotics in inhibiting biofilm formation may vary depending on the specific resistance gene carried by the CRKP strain. Additionally, absorbance measurements revealed that CRKP carrying *blaKPC* showed a notably higher absorbance after treatment with CZA than CRKP carrying *blaNDM*, further supporting the differential response of these strains to antibiotic treatment. These findings highlight the importance of considering the specific resistance mechanisms of CRKP strains when designing combination therapies, as the presence of different resistance genes can significantly influence the effectiveness of antibiotic treatments. Overall, these results underscore the potential of the CZA + ATM combination in overcoming biofilm-mediated resistance and enhancing bacterial cell disruption, providing a promising strategy for combating CRKP infections. However, the differential responses observed between *blaKPC*- and *blaNDM*-carrying strains emphasize the need for tailored therapeutic approaches based on the resistance profiles of the infecting pathogens.

Moreover, using qRT-PCR, the expression levels of genes encoding biofilm-associated proteins (*LuxS*, *mrkA*, *wbbM*, *pgaA*, and *wzm*) were reduced in both the single antibiotic group and the combined antibiotic group. Notably, in CRKP carrying the *blaKPC* resistance gene, the expression levels of the *luxS* and *wzm* genes were significantly lower after treatment with the ATM + CZA combination compared to treatment with a single antibiotic alone (*p* < 0.01). Similarly, in CRKP carrying the *blaNDM* resistance gene, the expression levels after treatment with the ATM + CZA combination were approximately 3 to 5 times lower than after treatment with CZA alone (*p* < 0.01). Notably, the combination of ATM and CZA induced the most pronounced reduction, with gene expression levels decreasing by approximately 3 to 5 times (Figure 6). Among these genes, the downregulation of *luxS* and *wzm* was particularly striking, suggesting their critical roles in mediating biofilm formation and stability. The *luxS* gene encodes a key enzyme in the quorum sensing (QS) system, which regulates bacterial communication and biofilm development. Its downregulation likely disrupts QS signaling, impairing the ability of CRKP to coordinate biofilm formation and virulence factor production. Similarly, *wzm* encodes a component of the extracellular polymeric matrix, which is essential for maintaining biofilm structure and resilience. The significant reduction in wzm expression observed in our study likely weakens the biofilm matrix, making CRKP more susceptible to antibiotic penetration and immune clearance. These findings provide new insights into the mechanisms by which CZA and ATM, particularly in combination, inhibit biofilm formation in CRKP. By targeting both the QS system (*luxS*) and the structural integrity of the biofilm (*wzm*), the combination therapy not only reduces biofilm production but also enhances the susceptibility of CRKP to antimicrobial agents. This dual mechanism of action may explain the superior efficacy of the combination therapy observed in our study and highlights its potential for treating refractory CRKP infections, which are often associated with biofilm-mediated resistance.

## 5 Conclusion

In conclusion, the combined use of ATM and CZA demonstrated significant synergistic bactericidal effects against CRKP strains producing various enzyme types, with the most notable effects observed against serine-producing strains. The combination also effectively disrupted CRKP cell structures and inhibited biofilm formation, offering additional options and theoretical evidence for treating difficult-to-manage infections caused by CRKP in clinical settings.

## Data Availability

The original contributions presented in the study are included in the article/Supplementary material, further inquiries can be directed to the corresponding authors.
